# Biosynthesized selenium nanoparticles: characterization, antimicrobial, and antibiofilm activity against *Enterococcus faecalis*

**DOI:** 10.7717/peerj.11653

**Published:** 2021-06-30

**Authors:** Sanjay Miglani, Nobuyuki Tani-Ishii

**Affiliations:** 1 Department of Conservative Dentistry & Endodontics, Faculty of Dentistry, Jamia Millia Islamia University, Delhi, India; 2 Department of Pulp Biology and Endodontics, Graduate School of Dentistry, Kanagawa Dental College, Yokosuka, Kanagawa, Japan

**Keywords:** Dentistry, Endodontics, Selenium nanoparticles, *Enterococcus faecalis*, Calcium hydroxide, Chlorhexidine gluconate, Sodium hypochlorite

## Abstract

**Background:**

Control over microbial growth is a crucial factor in determining the success of endodontic therapy. *Enterococcus faecalis* is the most resistant biofilm-forming species leading to endodontic failure. Hence, the current researches are directed towards discovering materials with superior disinfection properties and lesser cytotoxicity. This study aimed to synthesize and characterize biogenically produced Selenium Nanoparticles, and to evaluate the antimicrobial and antibiofilm efficacy, against *Enterococcus Faecalis*, for the following test groups: Group I: Distilled water (control), Group II: SeNPs (1 mg/ml), Group III: Calcium hydroxide (1 mg/ml), Group IV: 2% Chlorhexidine gluconate (CHX), Group V: 5.25% Sodium hypochlorite (NaOCl).

**Materials and Methods:**

Selenium nanoparticles were derived using fresh guava leaves (*Psidium guajava*) and were characterized. The antibacterial efficacy against *E. faecalis* was evaluated by agar well diffusion method. The antibiofilm efficacy of the test groups was observed by viable cell count, antibiofilm assay, and Anthrone and Bradford’s tests. The morphology of the biofilms was analysed using the Scanning Electron Microscope and Fourier Transform Infrared spectroscopy.

**Results:**

Antibacterial and antibiofilm efficacy of all tested solutions showed superior antibacterial and antibiofilm efficacy when compared to the control group. Overall, SeNPs (Group II) was the most effective against *E. faecalis* biofilm, followed by NaOCl (Group V), CHX (Group IV), and Ca(OH)_2_ (Group III).

**Conclusion:**

Biogenically produced SeNPs emerged as a novel antibacterial and antibiofilm agent against *E. faecalis*. This nano-formulation demonstrates the potential to be developed as a root canal disinfectant combating bacterial biofilm in endodontics after the results have been clinically extrapolated.

## Introduction

Endodontics is a branch of dentistry that deals with the diseases and treatment of tissues inside the roots of a tooth. The success of endodontic therapy depends on many factors, and amongst them, cleaning and shaping of the root canals and control over microbial growth are the most crucial factors. The types of bacteria in the endodontic space can be either facultative anaerobes or aerobes and some could be resistant species. *Enterococcus faecalis* is one of the main microorganisms associated with endodontic failures (Dioguardi et al., 2019). Its resistance to normal disinfection protocols is incurred due to its ability to form a biofilm, grow in resistant environments without oxygen, sustain in pH as alkaline as 11.5 and in temperatures as high as 60 °C, to overpower lymphocytes action, to grow in areas difficult to reach by instrumentation, due to its ability to express genes and activate different metabolic pathways under stress conditions (Jhajharia et al., 2015; Prada et al., 2019).

“A biofilm is an assemblage of microbial cells that is irreversibly associated (not removed by gentle rinsing) with a surface and enclosed in a matrix of primarily polysaccharide material” (Donlan, 2002). The current disinfection strategies are based on the effective use of disinfectants like Sodium hypochlorite (NaOCl), Chlorhexidine gluconate (CHX), Calcium hydroxide (Ca(OH)_2_), etc. for the elimination of microbes and their biofilms in the root canal system. However, a failure rate of 15–32% persists for primary root canal treatment due to several reasons (Prada et al., 2019; Ng et al., 2007).

To conquer the shortcomings of current disinfection strategies, many novel materials like nanomaterials are being tested for their antimicrobial and antibiofilm efficacy. Nanoparticles (NPs) that fall in the range of 1–100 nm, have a greater surface area, charge density, chemical reactivity, ability to interact with the bacterial cells, and thus enhanced antimicrobial activity due to the generation of free metal ion toxicity or reactive oxygen species (Khezerlou et al., 2018; Nisar et al., 2019). Due to their antibacterial properties, many nanoparticles are being tested to be used as irrigants, gels, medicaments, or additives to sealers and restorative materials in the field of endodontics (Shrestha & Kishen, 2016).

The synthesis of nanoparticles could be by physical, chemical, or by biological means. The biological method, also known as green synthesis, uses plants, fungi, and bacteria to synthesize nanoparticles and offers the advantage of being eco-friendly, less toxic, and economical as compared to other methods of production (Ingale & Chaudhari, 2013). Chitosan, bioactive glass, silver, quaternary ammonium polyethyleneimine nanoparticles (QPEINPs), zinc oxide are amongst the few nanoparticles which have been tried in endodontics (Samiei et al., 2016).

Selenium is an essential micronutrient in biological systems. Due to its antimicrobial, anticancer, antioxidant effects, SeNPs have many nanomedicine applications, and their cytotoxicity is lower than most commonly used silver nanoparticles (Hosnedlova et al., 2018; Chudobova et al., 2014). Selenium nanoparticles have been used in biomedical fields but their antimicrobial potential in endodontics yet to be explored. Amongst the chemical methods of synthesis, SeNPs are synthesized from selenite or selenous acid reduction by agents such as glutathione (GSH), hydrazine, sodium borohydride (NaBH_4_), stannous chloride (SnCl2), L-cysteine, ascorbic acid, sodium thiosulfate (Na_2_S_2_O_3_), and sodium dodecyl sulfate (SDS) (Stroyuk et al., 2008). Since, chemical methods are expensive, not ecofriendly, and may subject the particles to photo corrosion, greener methods of synthesis are sought after. Various plants and microbes have been used for the biological synthesis of Selenium nanoparticles (Murugesan, Nagaraj & Sunmathi, 2019; Piacenza et al., 2017). This study aimed to characterize biogenically produced Selenium nanoparticles, derived from fresh guava leaves (*Psidium guajava*), and evaluate its antimicrobial and antibiofilm efficacy against *Enterococcus faecalis* in comparison with Calcium hydroxide, 2% Chlorhexidine gluconate, 5.25% Sodium hypochlorite, and distilled water (control).

## Materials and Methods

The study proposal was approved by the Institutional Internal Research & Review Committee (Protocol No. FOD/IRRC/24/2019/F/11092019).

### Chemicals

Guava leaves (*Psidium guajava*) were gathered from the university campus. Sodium selenite salt (Sigma Aldrich, Bangalore, India), Blood Agar (Base) (Merck Mumbai, India), Defibrinated Sheep Blood (Thermo Fisher Scientific, Mumbai, India), 2% Chlorhexidine gluconate (Cerkamed, Stalowa Wola, Poland), 5.25% Sodium hypochlorite (Cerkamed, Stalowa Wola, Poland), and Calcium hydroxide (Prevest Denpro, Jammu, India) were used in the study. The rest of the chemicals used were of scientific grade.

### Biosynthesis of SeNPs

Biosynthesis and purification of selenium nanoparticles were carried out as described earlier (Alam et al., 2019). In brief, the guava leaves (10 g) that were plucked fresh from campus, were washed thoroughly with water. They were then cut and boiled in 100 ml of 60% ethanol for 2 min, followed by filtering through Whatman filter paper. The mixture was then diluted with distilled water to a 1:1 ratio. 900 ml of fresh aqueous sodium selenite (25 mM) was used to synthesize SeNPs by incubating it with 100 ml of guava leaf extract at 60 °C. It was then centrifuged at 13,280 RCF for 20 min to separate the SeNPs. Lastly, the pellet with SeNPs was washed with distilled water thrice and then air-dried.

### Characterization of nanoparticles

Characterization of the nanoparticles was done with the following techniques:

#### UV-Vis Spectrophotometer

The formation of Selenium nanoparticles (SeNPs) in the samples was supervised by gauging the UV-Vis spectra of the reaction medium. The UV-Visible spectroscopy of Selenium nanoparticles (SeNPs) was done using a Mecasys Optizen 3220 UV spectrophotometer.

#### Dynamic light scattering (DLS)

Spectroscatterer RiNA, GmbH class3B was used to measure the DLS of the samples.

#### Transmission electron microscopy

JEOL model JEM-2000FX instrument was used at an accelerating voltage of 200 kV to carry out the TEM analysis of SeNPs, as described earlier (Mazumder et al., 2020). Elemental analysis was done by an EDX (Model EVO-40; ZEISS, Jena, Germany) spectrum by placing SeNPs on a carbon-coated copper grid.

#### X-ray diffraction

XRD pattern was recorded on Bruker D8 advance diffractometer, over a wide range of Bragg angles (20° ≤ 2θ ≤ 80°), using Ni-filtered Cu-Kα X-rays of wavelength (λ) = 1.54056 Å. The raw data obtained, at the scanning rate of 0.05°/s, and subjected to the background correction and Kα2 reflections were removed using a normal stripping procedure.

### Antibacterial activity

#### Microorganism, culture conditions, and test groups

Bacterial strains were procured from Microbial Type Culture Collection (MTCC), Institute of Microbial Technology (Chandigarh, India). *Enterococcus faecalis* (MTCC 439) were cultured in luria broth and blood agar base with 5% defibrinated sterile sheep blood. Cells were maintained at 37 °C. The antimicrobial and antibiofilm efficacy against *Enterococcus faecalis* were evaluated for the following test groups: Group I: Distilled water (control), Group II: Selenium nanoparticles (1 mg/ml), Group III: Calcium hydroxide (1 mg/ml), Group IV: 2% Chlorhexidine gluconate (CHX), Group V: 5.25% Sodium hypochlorite (NaOCl).

### Minimum inhibitory concentration (MIC)

The microdilution method as reported previously, using 96-well microdilution plates, was followed for determining the MIC values to evaluate the antimicrobial activity of SeNPs (Wikler, 2006).

#### The agar diffusion test or Bauer–Kirby test

The antibacterial activity of different test groups (Group I–V) was evaluated against *Enterococcus faecalis* by the agar diffusion method, according to the standard protocol (Bauer et al., 1966). Fresh cultures (0.2 ml) of bacterial strains were inoculated into 5 ml of sterile luria broth separately and incubated for 3–5 hr to standardize the culture to McFarland standards (10^6^ CFU/ml). A total of 100 µl of revived cultures were added on a blood agar base with 5% defibrinated sterile sheep blood and poured on three replicate plates. Five paper discs (6 mm), each saturated with one of the test solutions were placed on the agar plates. The paper discs were saturated with one of the test solutions: 10–40 μl of graded concentration of SeNPs, 20 μl of 5.25% NaOCl, 20 μl of 2% CHX, 20 μl of Ca(OH)_2_ (1 mg/1 ml) and 20 μl of Distilled water as described earlier (Davis, Maki & Bahcall, 2007). All the experiments were performed thrice in triplicate.

### Antibiofilm activity

#### Antibiofilm assay

The antibiofilm activity was studied against *Enterococcus faecalis*. For biofilm formation, the cells were cultured in luria broth. In this luria broth, 0.2 ml of fresh bacterial cell cultures were inoculated and incubated at 37 °C to standardize the culture to McFarland standards (10^6^ CFU/ml). The cell culture (10 ml) was then treated with different test groups (Group I–V) and incubated at 37 °C in a shaker-incubator, kept at 180 RPM. A total of 500 μl of 1 mg/ml SeNPs, 500 μl of the rest of the test group were taken, without altering the concentration as received. The control bacterial cell culture group was incubated for the same time without any treatment. The biofilm formation was monitored visually in all the incubated cultures for 48 hrs. Later, the biofilms were centrifuged and washed with 1× PBS buffer thrice. The biofilms in both control and test groups were stained with crystal violet-1% (CV) and kept for 10 min. After incubation, the biofilm was washed several times with distilled water to remove the free dye. Finally, the CV infused decolouring solution was transferred to a clean 96 well plate with appropriate blanks (biofilms without any treatment) to be assessed for absorbance at 530–600 nm, with a Multiskan™ FC Microplate reader (O’Toole, 2011; Molobela, Cloete & Beukes, 2010).

$${\text{Percentage reduction of biofilm  =  }}[({\rm{C - B}}){\rm{ - }}({\rm{T - B}})){\rm{/}}({\rm{C - B}})] \times 100\% $$

where: B denotes, the average absorbance per well for blank (no biofilm, no treatment); C denotes the average absorbance per well for control wells (biofilm, no treatment) and T denotes the average absorbance per well for treated wells (biofilm and treatment).

#### Viable cell count

Viable cell count was analysed during the process of formation of the biofilms. Five ml (10^6^ CFU/ml) of the bacterial cell was used for biofilm formation (as described in “Antibiofilm Assay”). A total of 500 μl of 1 mg/ml SeNPs, 500 μl of the rest of the test group were taken, without altering the concentration as received, added to the bacterial sample. 2.5 ml of aliquot was taken out at different intervals of time (0 hrs, 24 hrs, 48 hrs) and optical density was analysed at 600 nm. To separate the biofilms from the aliquot, the sample was continuously vortexed for 2 min at a slow speed, which leads to the settling down of biofilms. One ml of aliquot was taken from the supernatant and absorbance was taken to measure the viable cells. The experiments were performed in three replicates and the result presented was the average of the three replicates.

#### Characterization and morphological analysis of biofilm

Biofilm formation took 48 hr, the test groups were mixed with cell culture that was kept for biofilm formation. After 48 hr samples for both FTIR and SEM were taken and further processing was carried for sample analysis. The carbohydrate and protein concentrations of treated and untreated biofilms were analysed by Anthrone and Bradford assay method, respectively (Bradford, 1976; Dreywood, 1946), and the reduction in their content was measured by Fourier Transform Infrared spectroscopy. The spectrum of the treated and untreated 48 hr old biofilm of *E. faecalis* was analysed on a Perkin–Elmer FTIR spectroscopy using KBr pellets. Biofilm materials were powdered and added to KBr to form pellets. To obtain a good spectra, 32 scans were taken in the frequency range of 600–4,000 cm^−1^ at a 4 cm^−1^ resolution. The morphological changes in biofilms after the treatment with different test groups (Group I–V) were investigated by using a Zeiss EVO 40 (Oberkochen, Germany) microscope at 20 kV. Samples for SEM were prepared as described by an earlier study (Mazumder et al., 2019).

### Statistical analysis

The result presented for all the antibacterial and antibiofilm assays is the mean from three replicates ± SD. The ANOVA test with repeated measurements and Student paired ‘t’ test was conducted to analyse significant differences. Statistical significance was taken as *p* = <0.05. The data were analysed by SPSS statistical software version SPSS 24.0.

## Results

### Characterization of SeNPs (Figs. 1 & 2)

**Figure 1:**
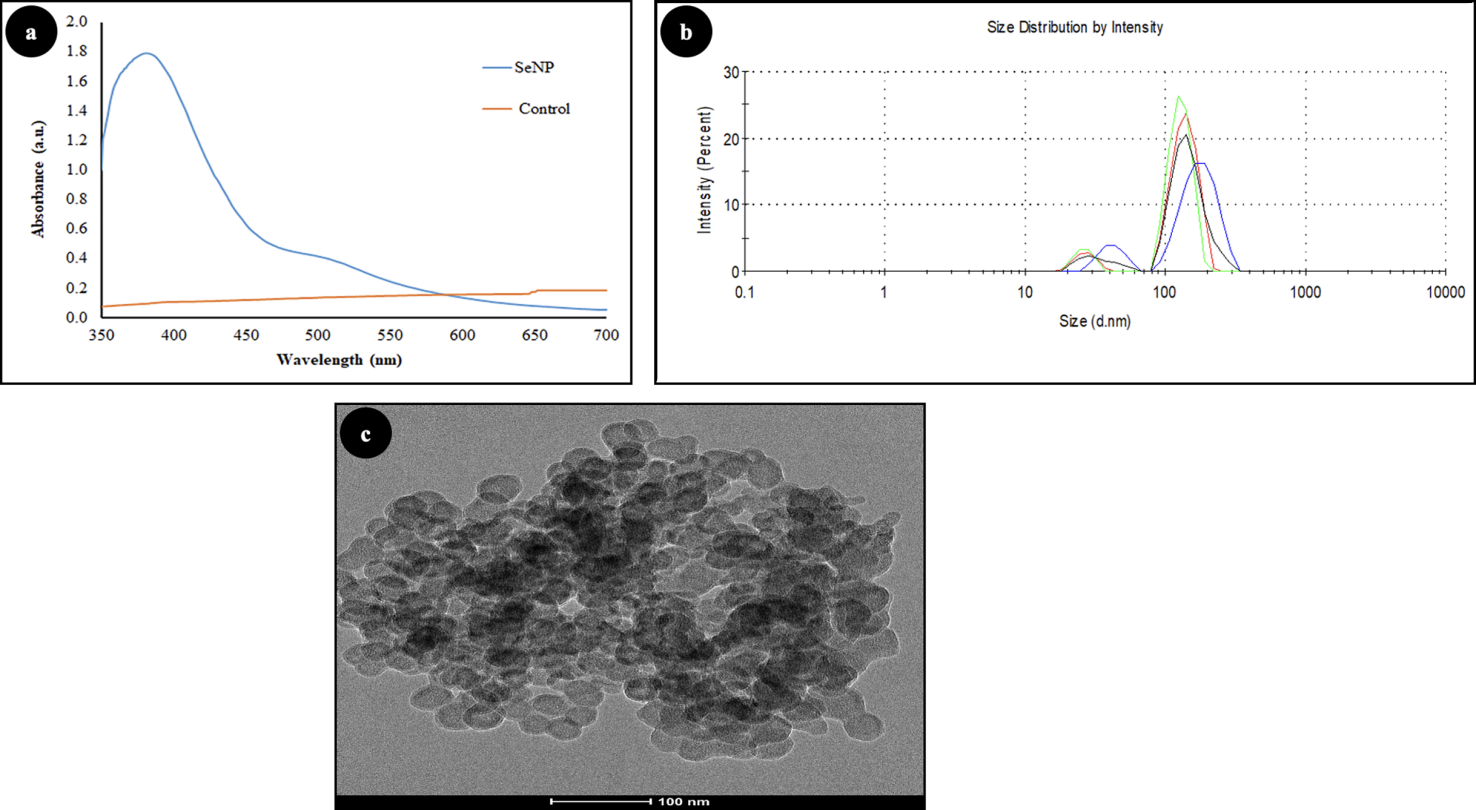
Characterization of SeNPs: (A) Ultraviolet spectroscopy, (B) dynamic light scattering and (C) transmission electron microscopy.

**Figure 2:**
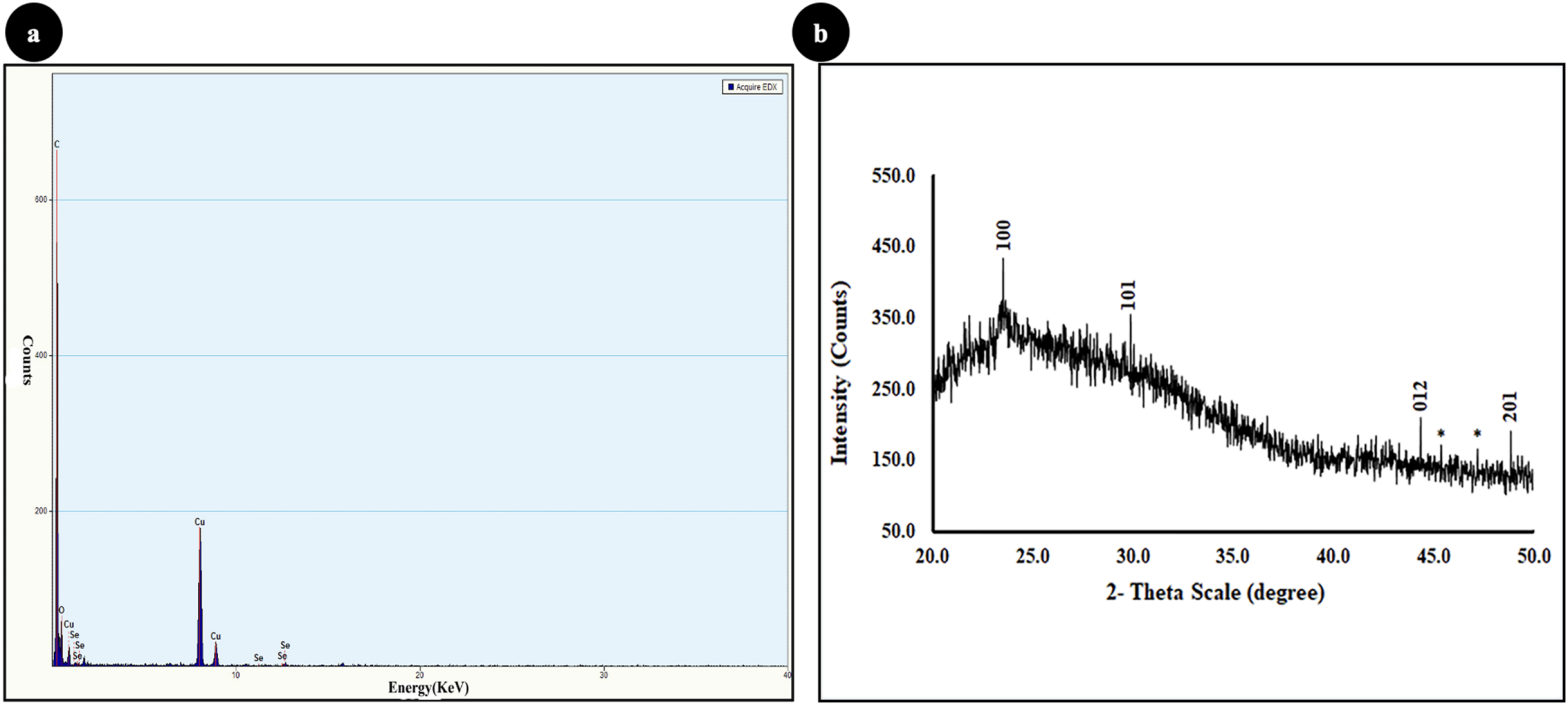
Characterization of SeNPs: (A) Energy-dispersive X-ray spectroscopy and (B) X-ray powder diffraction (XRD).

The formation of SeNPs was confirmed with ultraviolet (UV)-visible spectroscopy, in which the strong absorbance peak was observed at 388 nm (Fig. 1A). Control represents guava leaf extract in distilled water. The dynamic light scattering technique was carried out to measure the hydrodynamic radius and stability of SeNPs. The observed size in DLS was in the range from 40–150 nm (Fig. 1B). The particle size distribution of the selenium nanoparticles was determined by the polydispersity index (PDI), and PDI was found to be 0.30, suggesting a narrow size distribution of SeNPs. Zeta potential, which indicates the stability of nanoparticles and their ability to adhere to cell membranes, was found to be −60(mV), which indicates excellent stability of the colloidal dispersion, and a negative charge indicates a good adherence potential to a positively charged component of cell membranes. The particle size and surface morphology of the SeNPs were further confirmed with the help of TEM which revealed the particles were spherical and ranged from 30–50 nm (Fig. 1C). The EDX profile showed a strong Se signal (Fig. 2A), which suggests SeNPs obtained were of high purity and were crystalline in nature. The crystalline nature and purity of nanoparticles were also determined using powder X-ray diffraction technique (Fig. 2B). The peaks were observed at 23.3 (100), 29.6 (101), 43.5 (012), and 49.05 (201) which are in agreement with JCPDS file no. #73-0465. The biomolecules present in the plant leaf extract could have caused the unassigned peaks (*) in the XRD data.

### Antibacterial and antibiofilm efficacy

The antibacterial property of SeNPs along with different test conditions in solid media was evaluated by disk diffusion assay. The results presented are the mean from three replicates (Table 1). The mean zone of inhibition (mm) was lowest in Ca(OH)_2_ (6.83), followed by CHX (13.00), NaOCl (14.67), and higher in different concentrations of SeNPs (11.33, 16.50, 21.00 and, 28.50). The differences in the mean zone of inhibition under different treatment conditions were significantly different (*p* < 0.05) against control. Guava leaf extract and the precursor salt i.e., sodium selenite did not show any zone of inhibition, which suggests that the antibacterial property was only due to the interaction of SeNPs with the bacterial cell, and not due to any other entities that were used during the synthesis procedure. The MIC_80_ of SeNPs against *E. faecalis*, was found to be at 25 μg/ml (Table 2).

**Table 1:** Disk diffusion assay of different test groups against *Enterococcus faecalis*.

Group	Test conditions	Concentrations (μl)	Zone of inhibition (mm) (Mean & SD)
1	Control	20	–
2	SeNPs (1 mg/ml)	10	11.33 (± 0.57)
		20	16.50 (± 0.50)
		30	21.00 ± (1.00)
		40	28.50 ± (0.50)
3	Ca(OH)_2_ (1 mg/ml)	20	06.83 ± (0.28)
4	CHX	20	13.00 ± (1.00)
5	NaOCl	20	14.67 ± (0.57)
6	Guava leaf extract	20	–
7	Sodium selenite (25 mM)	20	–

**Table 2:** MIC80 of biosynthesised SeNPs against *Enterococcus faecalis*.

Compounds	MIC_80_(μg/ml)
	*Enterococcus faecalis*
SeNPs	25
Guava leaf extract	–
Gentamycin	17

**Figure 3:**
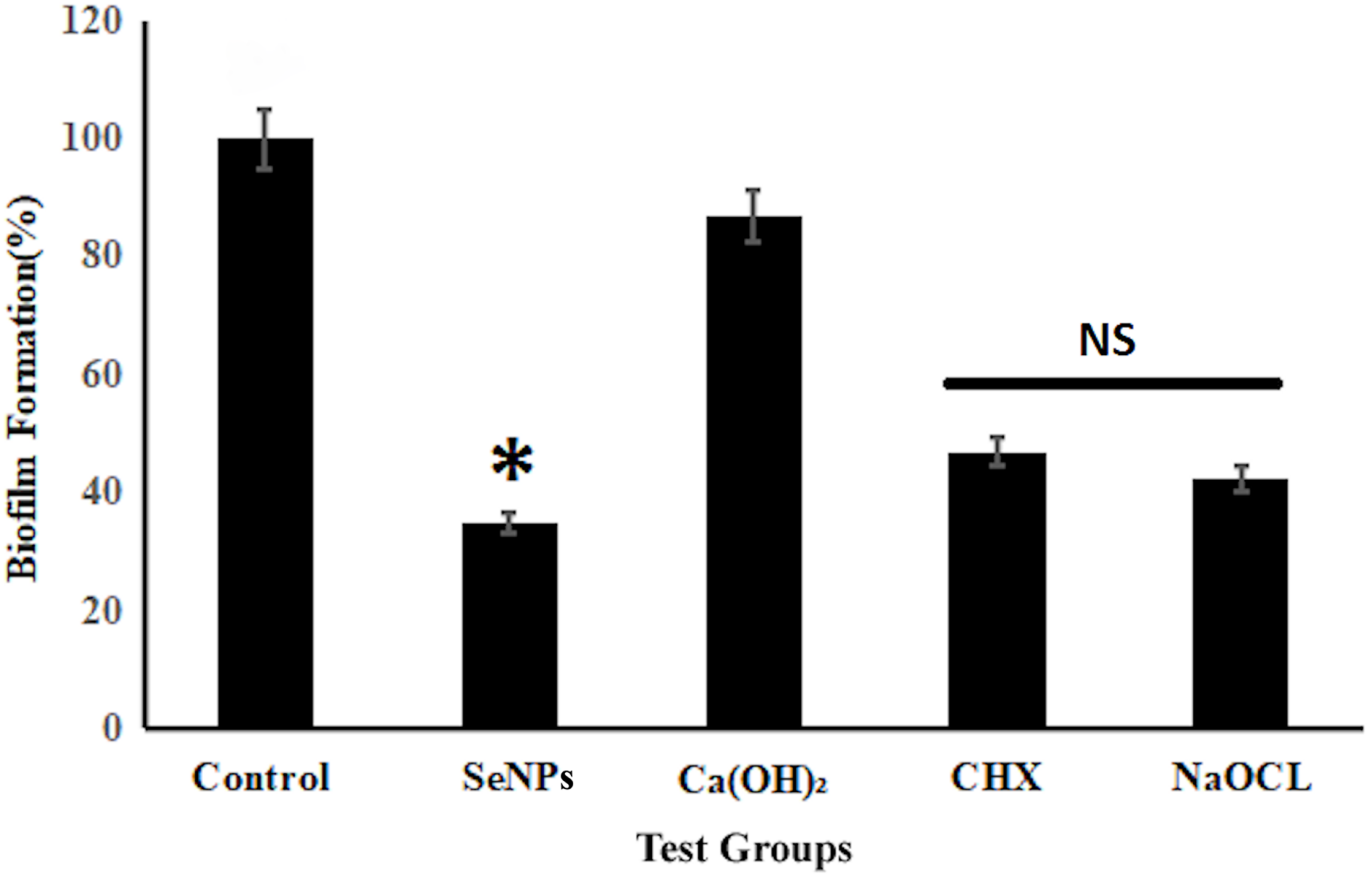
Antibiofilm efficacy of different test groups against *E. faecalis* biofilms. NS represents non-significant difference, whereas * represents significant difference as compared to control at *p* < 0.05.

The antibiofilm efficacy of the test groups was observed by Multiskan™ FC Microplate reader. The mean percentage decrease in growth of biofilms compared to control was highest in SeNPs, followed by NaOCl, CHX and was lowest in Ca(OH)_2_ (Fig. 3). The decrease in growth was highly significant (*p* < 0.001) in all test groups compared to control in antibiofilm assay test. It was observed that SeNPs, inhibited 65% growth of the biofilms (35% biofilm remaining).

**Figure 4:**
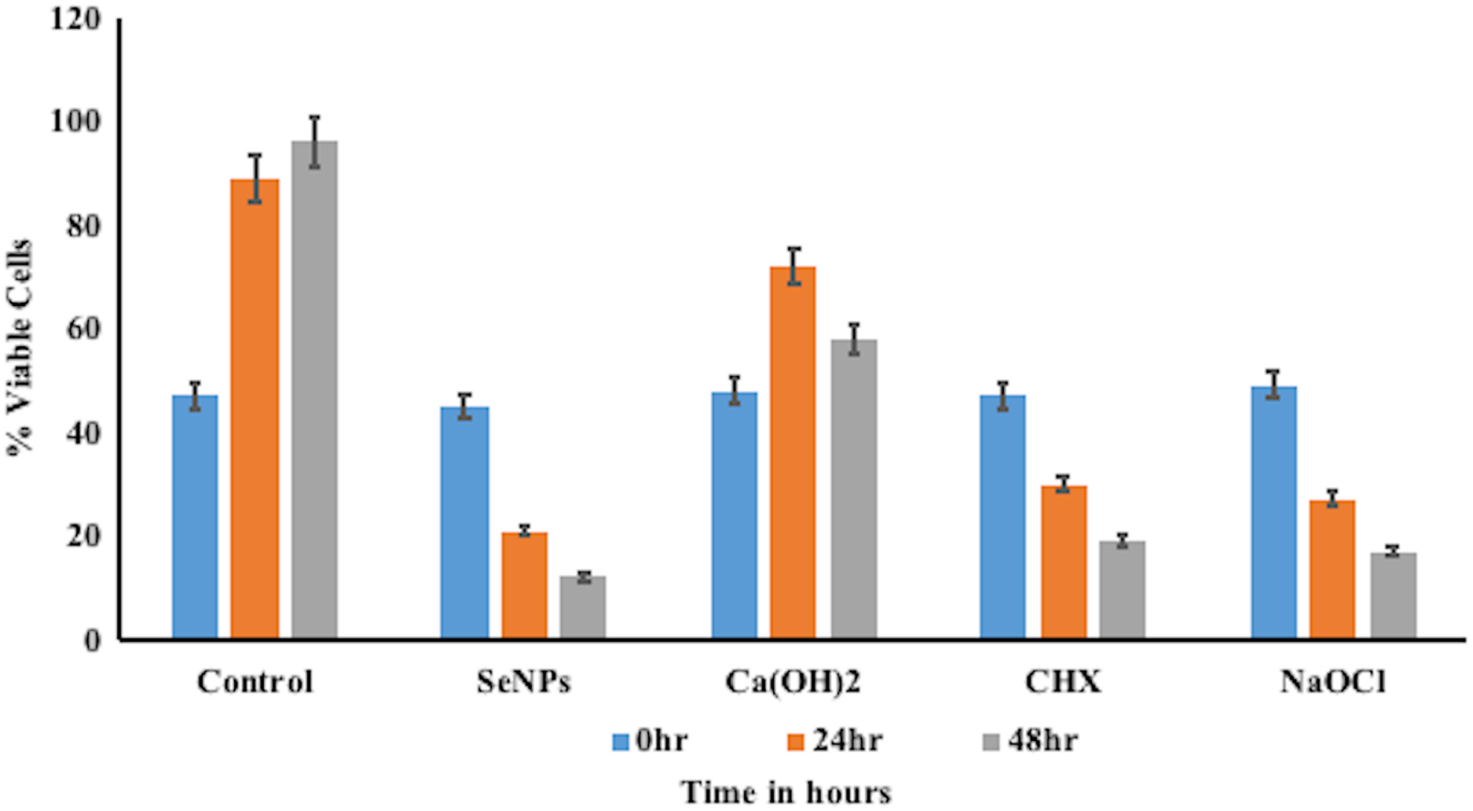
Viable cell percentage of different test groups against *E. faecalis*.

The ability of different groups to inhibit biofilm formation by *E. faecalis* was evaluated by counting the viable bacteria within the biofilm (Fig. 4). The percentage of viable cells at 24 hr, was highest in biofilms of Ca(OH)_2_ (72.20%) followed by CHX (30.03%), NaOCl (27.09%), and lowest was in SeNPs (21.38%), compared to control (89.06%). The mean percentage of viable cells was significant (*p* < 0.05) in all biofilms (SeNPs, Ca(OH)_2_, CHX, and NaOCl) compared to control (distilled water) at 24 hr. The percentage of viable cells at 48 hr, was highest in biofilms of Ca(OH)_2_ (58.10%) followed by CHX (19.15%), NaOCI (17.00%), and lowest was in SeNPs (12.13%), compared to control (96.16%). The mean percentage of viable cells was significant (*p* < 0.05) in all biofilms (SeNPs, Ca(OH)_2_, CHX, and NaOCl) compared to control (distilled water) at 48 hr.

The carbohydrate and protein content of the biofilms, with different test groups, were analysed by biochemical methods, Anthrone and Bradford tests (Table 3). The mean percentage reduction of carbohydrates contents in biofilm compared to control (31.33 (± 0.62)) was highest in SeNPs (08.37 (± 0.20), 73%), followed by NaOCl (10.23 (± 0.07), 67.30%), CHX (17.26 (± 0.08), 44.87%) and the lowest was in Ca(OH)_2_ (28.21 (± 0.07), 9.92%). The reduction was highly significant (*p* < 0.001) in all test groups compared to control under the Anthrone assay test. The mean percentage reduction of protein contents in biofilm compared to control (17.99 (± 0.21)) was highest in SeNPs (05.19 (± 0.17), 71%), followed by NaOCl (08.52 (± 0.04), 52%), CHX (10.29 (± 0.15), 43%) and lowest was in Ca(OH)_2_ (16.05 (± 0.20), 10.70%). The reduction of protein contents was highly significant (*p* < 0.001) in all test groups compared to control under the Bradford assay test. There was approximately 73% and 71% decrease in carbohydrate and protein content in the SeNPs group, respectively, as compared to control pointing towards a good antibiofilm efficacy.

**Table 3:** Carbohydrate and protein content of the *Enterococcus faecalis* biofilms treated with various test groups.

Anthrone assay biofilm		Bradford assay Biofilm
Carbohydrates (μg/mL) (Mean & SD)		Proteins (μg/mL) (Mean & SD)
Control	31.33 (± 0.62)	17.99 (± 0.21)
SeNPs	08.37 (± 0.20)	05.19 (± 0.17)
Ca(OH)_2_	28.21 (± 0.07)	16.05 (± 0.20)
CHX	17.26 (± 0.08)	10.29 (± 0.15)
NaOCl	10.23 (± 0.07)	08.52 (± 0.04)

**Figure 5:**
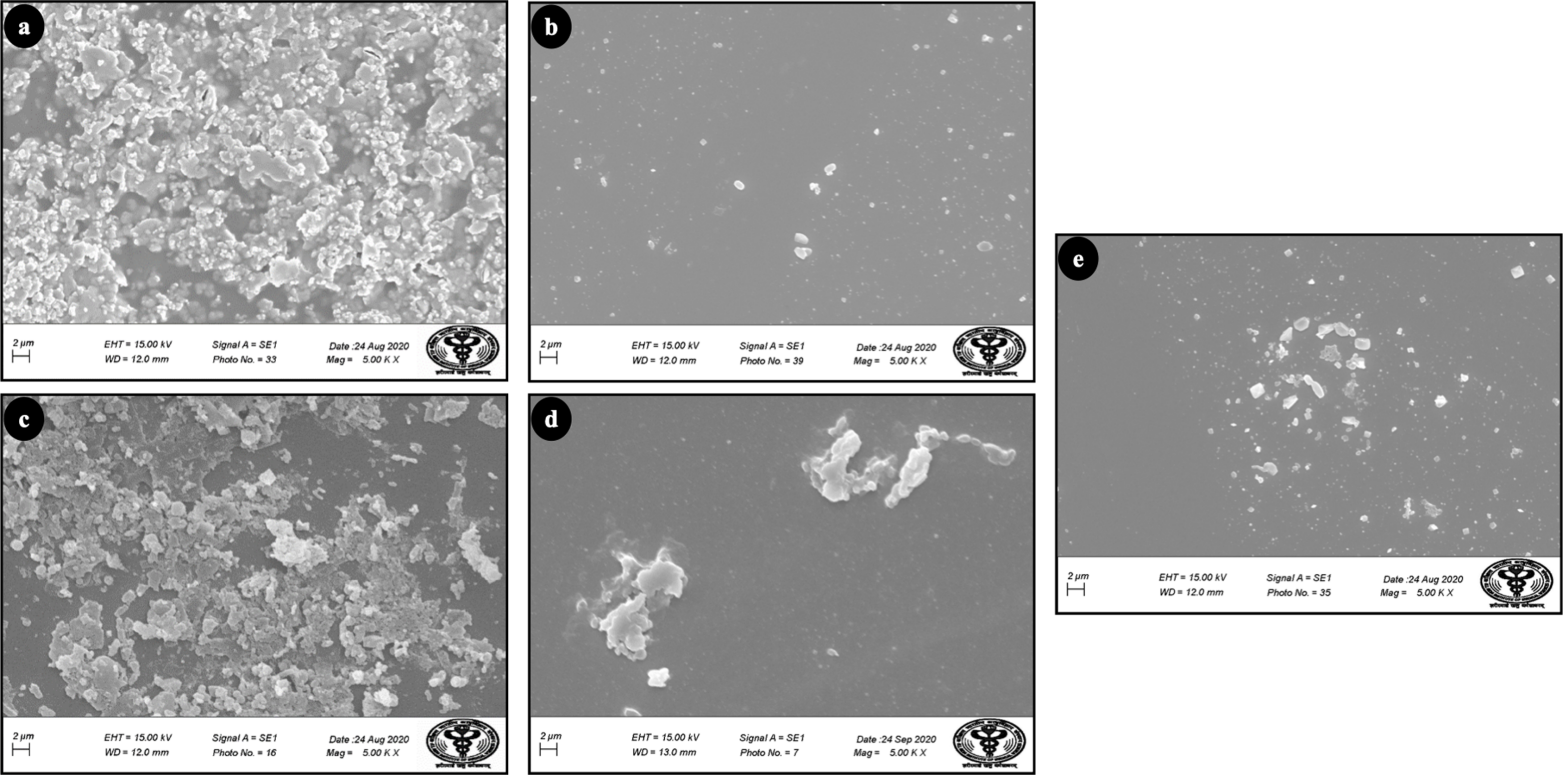
SEM image of *E. faecalis* biofilm (48 hr old) treated with different test groups: (A) Control, (B) SeNPs, (C) Ca(OH)_2_, (D) CHX and (E) NaOCl.

**Figure 6:**
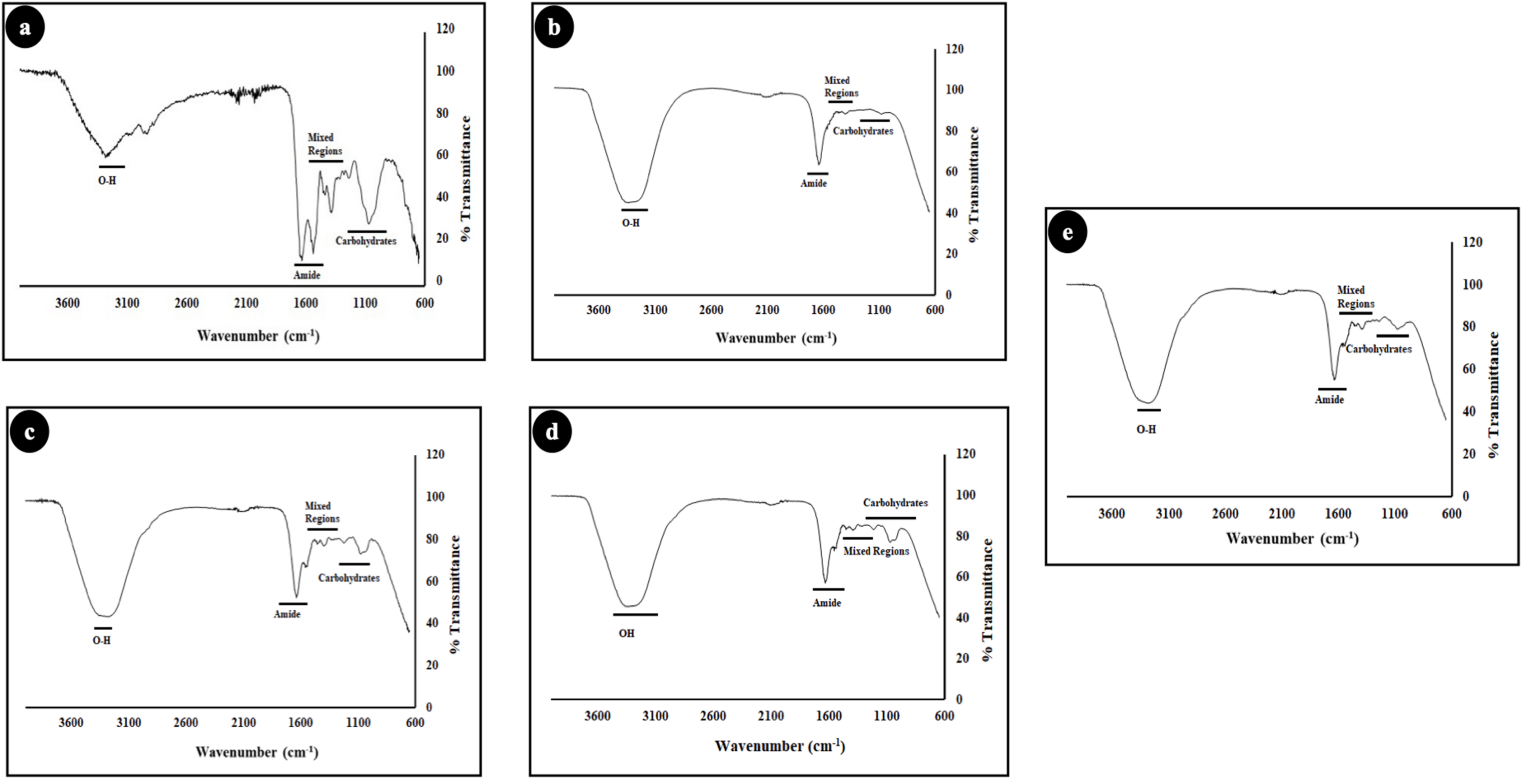
FTIR spectra of *E. faecalis* biofilm (48 hr old) treated with different test groups: (A) Control, (B) SeNPs, (C) Ca(OH)_2_, (D) CHX and (E) NaOCl.

The morphology of the biofilms was analysed using scanning electron microscopy (SEM), as shown in Fig. 5. The three test groups SeNPs, NaOCl and, CHX showed significant antibiofilm activity as compared to control and Ca(OH)_2_ which showed negligible antibiofilm efficacy as shown in Fig. 5A and 5C respectively. Further to validate the result of SEM & Anthrone and Bradford tests, Fourier-transform infrared spectroscopy (FTIR) was carried out (Fig. 6). The FTIR spectra of different vibrations in biofilms attribute to the presence of the proteins, mixed regions (ribose, deoxyribose, etc.), and carbohydrates and are mainly detected in the three following spectroscopic regions: 1,600–1,400 cm^−1^, 1,300–1,200 cm^−1^, and 1,200–1,050 cm^−1^. The range of peaks obtained from 880 to 1,200 cm^−1^ shows the carbohydrate content of the biofilm. Significant differences in peaks can be observed between the control and SeNPs treated biofilms in this region. Similar differences can be observed in the mixed regions between the control and SeNPs treated biofilms, which include 1,200–1,500 cm^−1^. The difference in the pattern of Amide I and Amide II peaks between the control and test conditions can also be observed. The difference in relative band intensities in spectra between the control and SeNPs treated biofilms signifies the change in carbohydrate and protein content in biofilms, which was further confirmed by SEM (Fig. 5); the images show that in the control group, the biofilm remains intact, whereas, it is scattered and degraded when treated with SeNPs. The differences in intensities of various test conditions as compared to the control biofilm result from the various quantitative contents of the above-mentioned compounds. The significant difference in intensities of various compounds of biofilms, when treated with SeNPs as compared to control, suggests the change in the structural integrity of the biofilms.

## Discussion

There can be many factors responsible for the failure of a root canal treatment and the persistence of bacteria in the canals is one of the leading reasons. Some bacteria would easily respond to conventional disinfection protocols, but there are a few which would be resistant and would lead to failure of endodontic treatment. *Enterococcus faecalis, Actinomycetes*, and *Propionibacterium propionicum* are the species of bacteria found to be most notorious, leading to persistent root canal infections (Dioguardi et al., 2019). Out of these, *Enterococcus faecalis* has been the main suspect in recurrent forms of apical periodontitis and thus is the most studied bacteria in the research to conquer the bacterial war in the canals.

Since nanoparticles are more efficient in their antibacterial properties due to reasons mentioned before, many are being experimented for their efficacy against this resistant *Enterococcus faecalis* which is also known to survive the most extremes and nutrient-free conditions. Chitosan, bioactive glass, silver, zinc oxide, quaternary ammonium polyethyleneimine are a few nanoparticles that have been tried in endodontics for their antibacterial properties (Waltimo et al., 2007; Bruniera et al., 2014; Shrestha et al., 2009; Guerreiro-Tanomaru et al., 2013; Cheng et al., 2012). Silver and ZnONPs have been tried against *E. faecalis* biofilms and 1% AgNPs and 26% ZnONPs had similar antibiofilm efficacy as compared to conventional irrigants (De Almeida et al., 2018). Chitosan nanoparticles have also been tried but they require prolonged treatment time for antibacterial effects (Shrestha et al., 2010).

Selenium, which is an essential trace element, in its nano-size has shown good antibacterial and anticancer properties (Khurana et al., 2019). Biosynthesized SeNPs, as compared to other means of synthesis, have shown low cytotoxicity towards normal cell lines making it a preferable material to be used in human studies (Alam et al., 2019; Zhang et al., 2005; Zhang, Wang & Xu, 2008). However, its antibacterial and antibiofilm efficacy against *E. facecalis*, so as to be used as a disinfectant in endodontics, has not been investigated and thus SeNPs were used in this study. The antibacterial action of these NPs is due to their ability to produce reactive oxygen species (ROS), depleting internal ATP, and disrupting membrane potential which leads to bacterial cell death (Huang et al., 2019). Due to their low toxicity and anticancer properties, their therapeutic benefits have been proven in many disorders like arthritis, nephropathy, diabetes, and cancer (Huang et al., 2013; Kumar et al., 2014; Li et al., 2011; Khurana et al., 2019). They have been found to be effective against many fungal and bacterial infections like *Trichophyton rubrum* (Yip et al., 2014), *Staphylococcus aureus* (Huang et al., 2016; Nguyen et al., 2017), and *E. coli* (Guisbiers et al., 2016). The antibiofilm potential of biogenically produced SeNPs has been proven against *P. aeruginosa* (Geoffrion et al., 2020), *Candida spp* (Cremonini et al., 2016) and *Proteus mirabilis* (Shakibaie et al., 2015). Selenium can be synthesized by physical means like laser ablation (Franzel et al., 2012), hydrothermal methods or ultraviolet radiation, chemical (Hosnedlova et al., 2018; Bartůněk et al., 2016; Zhang et al., 2004; Langi et al., 2010) methods like catalytic reduction, precipitation, acid decomposition, and biological methods using plants (Alagesan & Venugopal, 2019), fungi (Zare et al., 2013), or bacteria (Piacenza et al., 2017).

The green methods of synthesis, apart from being economical, have the advantage of not producing high temperature, pressure, acidic pH, and toxic by-products, and not requiring functionalization to produce hydrophilic or hydrophobic, conductive, or anticorrosive antimicrobial agents for biomedical applications, when compared to the physical and chemical methods. In this study, Selenium Nanoparticles were synthesized by aqueous sodium selenite (Na_2_SeO_3_) with an alcoholic extract of guava (*Psidium guajava*) leaf as reported in the study by Alam et al. (2019).

The SeNPs produced were of 30–50 nm size, which was much less as compared to the particle size mentioned in other studies using green synthesis. The particle size reported in other studies ranged from 80–100 nm (Geoffrion et al., 2020), from pulsed laser ablation in liquids, 29–195 nm (Shoeibi & Mashreghi, 2017) from *E. faecalis*, and 120 nm from *Providencia* sp.

The MIC_80_ of SeNPs against *E. faecalis*, was found to be at 25 μg/ml in this study, which is much lower than the one reported by Alam et al. by cytotoxic studies (Alam et al., 2019). Also, the MIC_99_ tested against *P. aeruginosa, S. aureus, E. coli and S. pyogenes* were found to be 125, 100, 100 and, 250 µg/ml of biosynthesized SeNPs which is much higher as compared to the present study (Srivastava & Mukhopadhyay, 2015). As in the present study low MIC value is observed, it suggests insignificant or no potential toxicity to humans or animal cells. The MIC_80_ concentration in this result is 25 μg/ml which is comparable to commercial antibiotic gentamycin having MIC_80_ concentration of 17 μg/ml (Table 2).

In this study, 4 test groups and 1 control group (distilled water) were evaluated for their antibacterial and antibiofilm efficacy. 2% Chlorhexidine gluconate (CHX) was used as it is recommended as an irrigant during root canal treatment due to its substantivity and its low cytotoxicity (Leonardo et al., 1999). A higher concentration of 5.25% was selected for Sodium hypochlorite (NaOCl) as at higher concentrations there is more undissociated hypochlorous acid (HClO) which is responsible for its antibacterial efficacy (De Almeida et al., 2018; Vianna et al., 2004). All tested solutions showed superior antibacterial and antibiofilm efficacy when compared to the control group. Overall, SeNPs were the most effective against *E. faecalis* biofilm, followed by NaOCl, CHX, and Ca(OH)_2_. The FTIR and SEM analysis also confirm the change in SeNPs treated biofilms as compared to control.

The results from earlier studies have been controversial for the two most used irrigants, viz. Sodium hypochlorite and Chlorhexidine for their antibacterial efficacy. A few have claimed that CHX is less effective as compared to NaOCl (del Carpio-Perochena et al., 2011), whereas others have shown both to be equally effective (Gomes et al., 2001). In this study, NaOCl performed slightly better than CHX. In a study conducted by De Almeida, 2% CHX and 5% NaOCl showed better antibiofilm efficacy when compared to AgNPs and ZnONPs, perhaps due to a short interaction period of 5 min (De Almeida et al., 2018). Though, biogenically produced AgNPs have shown to be equally effective as 2% CHX (Halkai et al., 2018).

In this study, SeNPs have demonstrated the potential to be used as an effective antimicrobial and antibiofilm agent for the disinfection of infected root canals. However, since the presence of organic media can influence the antibacterial and antibiofilm efficacy of NPs, further research is needed to verify these properties in the presence of organic media, at different concentrations, for different time exposures, and on *Enterococcus faecalis* extracted from an infected root canal. Also, the time required for disinfection, the mode of application (irrigant or medicament) should be further evaluated as it influences the interaction time that NPs would get with the bacteria, which could influence its efficacy. Apart from this, it has been documented that the Zeta potential produced or the charge that a nanoparticle carries also influences its antibacterial efficacy. In a previous study, it was shown that positively charged AgNPs showed better antibacterial efficacy than negative or neutral AgNPs (Abbaszadegan et al., 2015). In this study, though the SeNPs had a negative Zeta potential, the particles were fairly stable and showed superior properties to conventional irrigants. Though there are many studies which show nanoparticle with negative charges are better for preparing drug nanocarriers with maximized therapeutic efficacy and in vivo properties (He et al., 2010), and are also less toxic (Salvioni et al., 2017), further studies can be carried to evaluate the effect of differently charged SeNPs on *E. faecalis*.

Nanotoxicology has been a major concern since the advent of biomedical applications of Nanoparticles. Selenium Nanoparticles have 4-6 times lower toxicity as compared to selenium oxyanions, such as SeO3 −2 and SeO4 −2 (Zhang et al., 2005; Zhang, Wang & Xu, 2008). Severe toxicity due to SeNPs occurs only at higher doses. The median lethal dose (LD50) is 92.1 mg Se/kg for Nano-Se which is much higher than what was used in this study (1 mg/ml) (Zhang, Wang & Xu, 2008). In addition, SeNPs have been found to exhibit excellent anticancer and free radical scavenging properties. The biologically synthesized SeNPs have further reduced cytotoxicity and have been tested against various cell lines like Human non-small lung cancer cell line (Bharathi et al., 2020), HeLa (human cervical cancer) and SKOV-3 (human ovarian cancer) cells, (Kim et al., 2016) human keratinocytes (Matai et al., 2020), human breast cancer cells (MCF-7)(Ramamurthy et al., 2013). Its cytotoxicity has been found to be less than the most commonly used silver nanoparticle (Hosnedlova et al., 2018; Chudobova et al., 2014). Detailed literature on nano selenium, it’s reduced cytotoxicity, and various biomedical applications have been documented by Hosnedlova et al. (2018). The SeNPs used in our study were biologically synthesized with guava leaf extracts and their cytoxicity has been previously evaluated using a cancerous cell line HepG2 and normal cell lines CHO procells in a study done by Alam et al. (2019). Since the cytotoxicity of SeNPs is lower than most used silver nanoparticles (Hosnedlova et al., 2018; Chudobova et al., 2014), they offer promising potential in the field of endodontics, though the results need to be clinically extrapolated. This study could serve as a baseline to further explore the potential of SeNPs or its combinations, against other endodontic pathogens.

## Conclusion

Biogenically produced SeNPs have emerged as a novel antibacterial and antibiofilm agent against *E. faecalis*. This nano-formulation demonstrates the potential to be developed as a root canal disinfectant combating bacterial biofilm in endodontics after the results have been clinically extrapolated.

## Supplemental Information

10.7717/peerj.11653/supp-1 Supplemental Information 1 Raw data.

## Additional Information and Declarations

### Data Availability

The following information was supplied regarding data availability: The raw measurements are available in the Supplemental File.
